# Acute systemic 11-*cis*-retinal intervention improves abnormal outer retinal ion channel closure in diabetic mice

**Published:** 2012-02-07

**Authors:** Bruce A. Berkowitz, David Bissig, Priya Patel, Ankit Bhatia, Robin Roberts

**Affiliations:** 1 Department of Anatomy and Cell Biology, Wayne State University, Detroit, MI; 2 Department of Ophthalmology, Wayne State University, Detroit, MI

## Abstract

**Purpose:**

To test the hypothesis that in dark-adapted diabetic mice subnormal manganese uptake in the outer retina can be ameliorated with exogenous 11-*cis*-retinal intervention.

**Methods:**

Three groups were studied: age-matched controls and mice that had been diabetic for 3 months with and without acute, systemic 11-*cis*-retinal treatment administered 30 min before the manganese injection. Mice in each group were examined with manganese-enhanced magnetic resonance imaging (MEMRI) to assess central intraretinal manganese uptake and extraocular muscle manganese uptake. Bodyweights and glycated hemoglobin were determined.

**Results:**

Both diabetic groups had lower bodyweights and higher glycated hemoglobin levels relative to controls; no differences in these parameters between diabetic groups were noted. No substantial differences in muscle uptake were noted between any of the groups. Diabetes produced a subnormal intraretinal uptake of manganese; acute exogenous 11-*cis*-retinal significantly corrected only outer retinal uptake, although not to control levels.

**Conclusions:**

The present results provide for the first time evidence that raises the possibility of a critical role of 11-*cis*-retinal, a key participant of the visual cycle, in diabetes-evoked outer retinal dysfunction.

## Introduction

Vision begins in the photoreceptors. In the dark, healthy rod photoreceptor membranes are primarily depolarized via open cyclic guanosine monophosphate (cGMP)-gated ion channels. In the light, rhodopsin (which consists of opsin and covalently bound 11-*cis*-retinal) undergoes a *cis*-to-*trans* isomerization. Through a series of signal transduction steps, this isomerization normally results in closure of rod cGMP channels and hyperpolarization of rod membranes. However, in non-diabetic mouse models of visual cycle disease, regeneration of rhodopsin, due, for example, to reduced availability of 11-*cis*-retinal, is impaired leading to the following sequential chain of events: a persistent buildup of unbound opsin, cGMP hydrolysis, closure of ion channels, and chronic hyperpolarization of the photoreceptor membranes even in the dark [1]. Importantly, systemic 11-*cis*-retinal administration is effective at relieving such visual cycle lesions and maintaining photoreceptor membranes in a depolarized (i.e., open) state in the dark, consistent with the generation of sufficient rhodopsin to relieve opsin buildup and decrease cGMP hydrolysis [2].

Diabetic retinopathy, the leading cause of vision loss and blindness in patients under the age of 65 years old, is clinically considered a vascular disease. However, there is mounting evidence of neural retinal dysfunction before the appearance of vascular lesions [3–7]. Intriguingly, diabetes imparts overt retinal microvessel histopathology but largely leaves brain blood vessels unharmed [8]. This discrepancy between retinal and brain responses to diabetes has focused attention on a unique aspect of the retina compared to the brain: the photoreceptors. Patients can present with sensory neuronal pathophysiology linked with alterations in key aspects of vision including impaired dark adaptation [7,9]. Furthermore, focal changes in neuronal retinal function predict the onset of diabetic retinopathy with a sensitivity of 80% and a specificity of 74% [6,10]. To date, fundamental knowledge gaps exist with regard to how layer-specific neuroretinal lesions are produced by diabetes. Correlations have been found between diabetes and subnormal rhodopsin generation, reductions in the expression of genes involved with the phototransduction pathway, and with visual cycle protein levels [11–14]. These results suggest but do not prove the presence of visual cycle lesions in diabetic retinopathy. One approach to directly testing this idea is to intervene with 11-*cis*-retinal (a drug targeted to visual cycle defects) and determine if outer retinal ion dysfunction improves.

Previously, we demonstrated that genetically-induced, impaired 11-*cis*-retinal production in mice produced an abnormal outer retinal ion channel closure on manganese-enhanced magnetic resonance imaging (MEMRI) and that acute, exogenous 11-*cis*-retinal corrected this lesion [2]. In the present study, we asked the question: would acute treatment of dark-adapted diabetic mice with systemic 11-*cis*-retinal alter inner and outer retinal ion channel status as interrogated non-invasively with MEMRI? The results from the present experiments demonstrate that diabetes-evoked abnormal outer retinal ion channel closure can be significantly, if partly, improved with acute exogenous 11-*cis*-retinal.

## Methods

All animals were treated in accordance with the National Institutes of Health Guide for the Care and Use of Laboratory Animals, the Association for Research in Vision and Ophthalmology Statement for the Use of Animals in Ophthalmic and Vision Research, and Institutional Animal and Care Use Committee authorization. Animals were housed and maintained in normal 12 h:12 light-dark cycle laboratory lighting, unless otherwise noted.

### Groups

MEMRI was used to study different groups of male C57BL/6 mice (Jackson Laboratories, Bar Harbor, ME): control group (wild type (WT); n=9, weight range 30–36 g, age about 5 months), diabetic group (D; n=9, weight range 17–23 g, age about 5 months, diabetic for 3 months), and diabetic + 11-cis-retinal group (D+11cis; n=10, weight range 20−25 g, age about 5 months, diabetic for 3 months). 11-*cis*-retinal (approximately 25 mg/kg mouse), obtained through an NEI program, was constituted in a 10% ethanol and phosphate buffered saline and BSA solution, and administered as a 0.01 ml/g bolus intraperitoneal [15] 30 min before the manganese injection (see below).

Diabetes was induced in mice with starting weights of 16 to 20 g by streptozotocin (60 mg/kg; 10 mM citrate buffer [pH 4.5]) intraperitoneal injection once a day for 5 consecutive days. Bodyweight and blood glucose levels were monitored twice weekly. Insulin (neutral protamine Hagedorn), administered to mice as needed based on bodyweight and blood glucose levels but not more than twice weekly, allowed slow weight gain while maintaining hyperglycemia (blood glucose levels higher than 400 mg/dl). Mice that lost weight and/or had blood glucose levels greater than 600 mg/dl were given 0.2 units of Lilly Humulin N insulin. Normal rodent chow (Purina TestDiet 5001; Richmond, IN, which contains 11.2% fat, 26% protein and 62.7% carbohydrate) and water were provided ad libitum. Glycated hemoglobin was measured from blood collected after each MEMRI experiment (Glyco-Tek affinity columns, kit 5351; Helena Laboratories, Beaumont, TX). Blood was drawn from the left ventricle, after puncture, into a capillary tube and stored in an eppendorf tube with a small amount of heparin to prevent coagulation. The blood was kept in the refrigerator until analysis within one week following the MEMRI experiment.

### Outcome measures

The methodologies for measuring MEMRI parameters have been described in detail previously [3]. Briefly, for MEMRI, all mice were maintained in darkness for 16 to 20 h before manganese injection. All procedures (e.g., weighing, injecting 11-*cis*-retinal and MnCl_2_, anesthetic administration, and magnetic resonance imaging [MRI] examination) were performed under dim red light or darkness. MnCl_2_ was administered as an intraperitoneal injection (66 mg/kg) on the right side of awake mice. After this injection, mice were maintained in dark conditions for another 3.5 to 4 h. Immediately before the MRI experiment, mice were anesthetized with urethane (36% solution intraperitoneally; 0.083 ml/20 g animal weight, prepared fresh daily; Aldrich, Milwaukee, WI). MRI data were acquired on a 7T system (Clinscan; Bruker, Billeric, MA). Retinal partial saturation T_1_ data were acquired using a dual coil mode on a 7 T Bruker Clinscan system: Several single spin-echo (time to echo [TE] 13 ms, matrix size 160×320, slice thickness 600 μm) images were acquired at different repetition times [TRs] in the following order (number per time between repetitions in parentheses): TR 0.15 s (6), 3.50 s (1), 1.00 s (2), 1.90 s (1), 0.35 s (4), 2.70 s (1), 0.25 s (5), and 0.50 s (3). To compensate for reduced signal-noise ratios at shorter TRs, progressively more images were collected as the TR decreased. After the MEMRI examination, a final blood sample was obtained for glycated hemoglobin analysis, and the mice were humanely euthanatized.

### Data analysis

**Figure 1:**
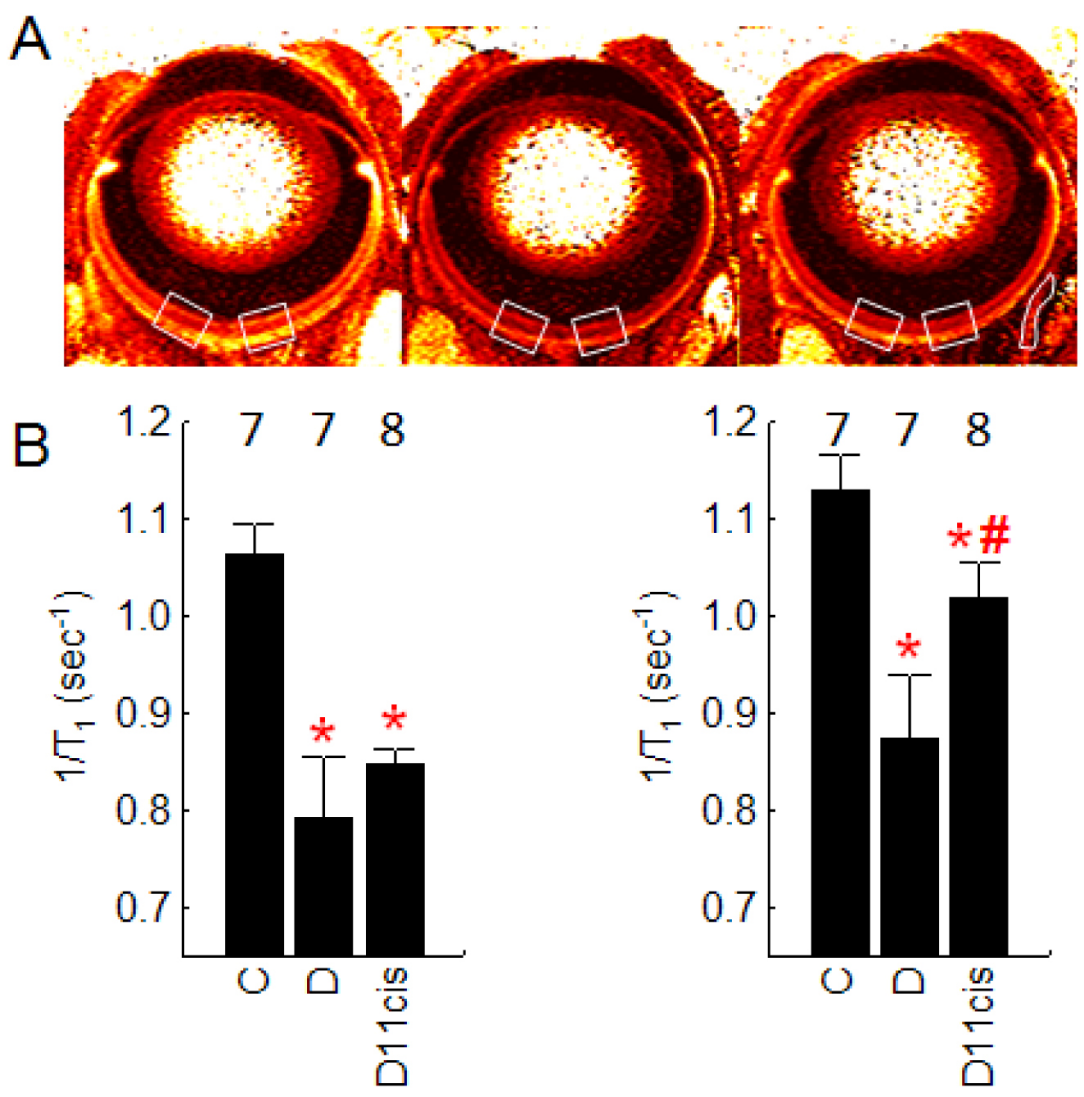
The inner and outer retina of diabetic mice take up less manganese than in controls – a pattern partially corrected in the outer retina upon treatment with 11-cis-retinal. **A**: Representative pseudo-color spin-lattice relaxation rate (1/T1) parameter maps of control (left), diabetic (middle), and diabetic treated with 11-cis-retinal (right) mice illustrate lower uptake in diabetics, and partial correction with 11-cis-retinal treatment. Note that 1/T1 values directly reflect manganese levels [16] and greater manganese uptake is indicated by brighter retinal reds. The central retinal regions analyzed in this study are indicated by the white boxes and the extraocular muscle region-of-interest analyzed herein is illustrated in the right-most image. **B**: Quantitative analysis of manganese uptake in inner (left graph) and outer retina (right graph) confirms the pattern suggested by the representative images above. * indicates a significant difference (p<0.05) from control mice values; # indicates a significant difference (p<0.05) from the diabetic mice values. The error bars represent standard deviations, and numbers above the bars represent the number of animals studied.

MEMRI data of central retinal data (±1 mm from the center of the optic nerve, see Figure 1A) were analyzed as follows: single images acquired with the same TR were first registered (rigid body) and then averaged. These averaged images were then registered across TRs. The same regions-of-interest as above were analyzed by calculating 1/T_1_ maps by first fitting to a three-parameter T_1_ equation (y=a + b*(exp(-c*TR)), where a, b, and c are fitted parameters) on a pixel-by-pixel basis using R (v.2.9.0, R Development Core Team [2009]). R: A language and environment for statistical computing. R Foundation for Statistical Computing, Vienna, Austria. ISBN 3–900051–07–0) scripts developed in-house, and the minpack.lm package (v.1.1.1, Timur V. Elzhov and Katharine M. Mullen minpack.lm: R interface to the Levenberg-Marquardt nonlinear least-squares algorithm found in MINPACK. R package version 1.1–1.). The reciprocal (1/T_1_) values directly reflect manganese levels [16]. On 1/T_1_ parameter maps, the inner and outer retinal division is observable in dark-adapted retinas, and pixels immediately anterior and posterior to this division were extracted as follows. On relaxation rate (1/T_1_) maps of dark adapted retina, the inner/outer retina division was observable (based on contrast generated by the differential amount of manganese taken up in inner and outer retina on these maps). We measured relaxation rates from one pixel layer located anterior and posterior of this division to sample inner and outer retina, respectively. In addition, central intraretinal 1/T_1_ profiles were examined in each mouse to ensure that the choice of inner / outer retinal division was appropriate and that the representative inner and outer retinal values were reasonable [17]. The 1/T_1_ of the same extraocular muscle group was measured in all groups to compare systemic handling of the injected manganese.

Note that only those animals that took up manganese above baseline (0.65 s^−1^) were included in the final analysis of retinal and extraocular muscle. In addition, the same extraocular muscle region-of-interest was either not present (i.e., out of slice) or was difficult to discern for some animals. For these reasons, note that the numbers of animals used in each measure were somewhat different.

### Statistical analysis

Comparisons of MEMRI retinal signal intensities were performed using a generalized estimating equation (GEE) approach [17,18]. GEE performs a general linear regression analysis using all of the pixels in each subject and accounts for the within-subject correlation between adjacent pixels. GEE was performed using the GENMOD procedure in SAS for Windows with the working correlation matrix set to autoregressive [1] and the scale parameter set to the Pearson χ^2^. The MEMRI extraocular muscle 1/T_1_ data, measured from a single region-of-interest and thus not subject to correlations between pixels, was consistent with a normal distribution and data analyzed with a two-tailed *t* test. In all cases, two-tailed p<0.05 was considered statistically significant.

## Results

### Systemic physiology

In this study, control mice bodyweights were 32.8±0.5 g (mean±SEM), and the glycated hemoglobin levels were 5.9±0.2%. Untreated diabetic mice had bodyweights of 19.3±0.9 g, and glycated hemoglobin levels of 13.2±0.5%. The 11-*cis*-retinal-treated diabetic mice bodyweights were 22.5±0.6 g, and their glycated hemoglobin levels were 12.9±0.3%. The difference in bodyweights and glycated hemoglobin levels were statistically different between diabetic mice and control mice (p<0.05), and there were no differences in bodyweight or glycated hemoglobin levels between treated and untreated diabetic groups.

### Manganese-enhanced magnetic resonance imaging

Extraocular uptake of manganese, an indicator of systemic loading of the injected manganese, was not statistically different (p>0.05) between the controls (n=6, 1.10±0.07 s^−1^, range 0.9–1.3 s^−1^), untreated diabetics (n=7, 1.02±0.06 s^−1^, 0.8–1.2 s^−1^), and treated diabetics (n=8, 1.10±0.03 s^−1^, 0.9–1.2 s^−1^). This demonstrates similar uptake and delivery of manganese in all groups.

Qualitative inspection of 1/T_1_ parameter maps suggested that diabetic mice had reduced uptake from controls, and that 11-*cis*-retinal treatment of diabetic mice partially restored central retinal uptake of manganese (Figure 1A). Quantitative analysis (Figure 1), demonstrated that the untreated mice that had been diabetic for 3 months had significantly lower manganese uptake in the inner and outer retina (66% and 53% of control, respectively; calculated after subtracting baseline 1/T_1_ of 0.65 s^−1^). Diabetic animals treated acutely with 11-*cis*-retinal maintained (p>0.05) reduced inner retinal uptake, but outer retinal manganese uptake was increased (p<0.05) to 23% of control (Figure 1B).

## Discussion

In this study, in dark-adapted diabetic mice in vivo, 11-*cis*-retinal treatment significantly, but partially, improved outer retinal manganese uptake without a discernable benefit on uptake in the inner retina. This is consistent with the highly selective role of 11-*cis*-retinal in the outer retina as the key light-sensing chromophore. Group differences in systemic handling of the injected manganese do not explain the variations in intraretinal uptake patterns because of the distinct intraretinal patterns noted above and the similar extent of muscle uptake between groups. Although 11-*cis*-retinal uptake, transport, and storage are complicated [19], and may be impaired by diabetes, the present results support some benefit of 11-*cis*-retinal supplementation on manganese-permeable outer retinal ion channels in a manner consistent with visual cycle biology [2]. We based the timing of the present studies on our previous work in rd12 mice in which 4 h between 11-*cis-ret*inal injection and MEMRI examination was adequate for documenting effective opening of outer retinal ion channels, even though all of the 11-*cis*-retinal was not likely to be available at this time [2,19]. Future studies are needed to determine if higher doses or longer duration of treatment with 11-*cis*-retinal would be more effective at further improving outer retinal function [19]. To further probe the molecular mechanisms underlying how exogenous 11-*cis*-retinal opened outer retinal ion channels, future studies will measure retinal levels of 11-*cis*-retinal or 11-*trans*-retinal, as well as systemic vitamin A levels [20]. Nonetheless, the present results raise the possibility, for the first time, of a critical contribution of 11-*cis*-retinal to a diabetes-produced outer retinal lesion in vivo.

MEMRI non-invasively measures, in continuously dark-adapted awake and freely moving mice, a history of neuronal activity based on the extent of retinal layer-specific accumulation of a calcium ion surrogate, manganese ion [4,17,21]. Importantly, the MEMRI-related procedures require little to no light, and this greatly facilitates evaluating the efficacy of 11-*cis*-retinal, an extremely light-labile compound.

Previously, we found that preventing oxidative stress in diabetic mice or rats corrected the subnormal intraretinal uptake on MEMRI [3,4]. Intriguingly, Anderson et al. reported that elevated oxidative stress per se could reduce how much rhodopsin is regenerated from exogenously added 11-*cis*-retinal by 40%–50%; our present results also found a partial benefit on outer retinal channels (consistent with partial rhodopsin regeneration from exogenous 11-*cis*-retinal) [22]. It is not yet clear how retinal oxidative stress (due to diabetes) and rhodopsin regeneration are mechanistically linked. One suggestion is that diabetes induces a more acidic condition within the rod photoreceptors that causes a delay in rhodopsin regeneration [23]. However, direct pH measurements in diabetic animals did not seem to support this notion [24,25]. Diabetes clearly alters mitochondrial function, and this could reduce the energy available for keeping the cGMP channels open in the dark [26,27]. The present results might not support this idea: 11-*cis*-retinal supplementation partly restored manganese uptake indicating that enough energy was available to maintain open channels, at least to some degree. These data are consistent with our previous finding that retinal ATP levels are similar between diabetic and controls [28]. Alternatively, it is possible that oxidative stress alters, for example, RPE65 activity and thus reduces 11-*cis*-retinal synthesis in diabetic mice. Oxidative stress is reported to cleave RPE65 in retinal pigment epithelium cells although the functional significance of the cleavage product is unknown [29]. In addition, systemic vitamin A may have been generally less available to the diabetic retina [20]. Although additional future studies are needed to address the above possibilities, regardless of mechanism, the present findings are consistent with acute, systemic 11-*cis*-retinal treatment at least partly improving a key aspect of a visual cycle lesion in the diabetic mouse model.

The results of this study, and the above considerations, provide for the first time evidence supporting a role of 11-*cis*-retinal in a diabetes-evoked functional defect specifically in the outer retina in vivo.
